# Effects of a fixed nurse team in the orthopaedic surgery operating room on work efficiency and patient outcomes: a propensity score-matched historically controlled study

**DOI:** 10.1186/s12912-022-01027-5

**Published:** 2022-09-06

**Authors:** Huaying Zhong, Limin Zhou, Shaoling Liao, Jing Tang, Liqun Yue, Meizhen Mo, Yiyue Zhong

**Affiliations:** 1 Department of Operating Room, Affiliated Hospital of Guangdong Medical University, No. 57 People Avenue South, Zhanjiang, 524001 China; 2 Department of Nursing Research, Affiliated Hospital of Guangdong Medical University, No. 57 People Avenue South, Zhanjiang, 524001 China; 3 Department of Anaesthesiology, Affiliated Hospital of Guangdong Medical University, No. 57 People Avenue South, Zhanjiang, 524001 China

**Keywords:** Nursing administration research, Nursing quality, Operating room nursing, Orthopaedic surgery, Fixed nurse team

## Abstract

**Background:**

The work value of operating room (OR) nurses is directly reflected in nursing quality. However, evaluating the work value of these nurses has not been sufficiently investigated. This study evaluated the effects of a fixed nurse team (FNT) in an orthopaedic surgery OR on work efficiency and patient outcomes.

**Methods:**

A propensity score-matched historically controlled study conducted from 1 July 2015 to 30 June 2018 was used to investigate the difference in nursing quality between an FNT period and a non-FNT period in the orthopaedic surgery OR at a tertiary care hospital in China. The primary outcome was surgical site infections (SSIs) during in-hospital visits, and as a secondary outcome, other nursing-sensitive quality indicators were assessed with historically controlled data. A multifactor logistic regression model was constructed to examine the primary outcome differences between the FNT and non-FNT periods before and after propensity score matching.

**Results:**

In total, 5365 patients and 33 nurses were included in the final analysis. The overall SSI rate was 2.1% (110/5365; the non-FNT period 2.6% [64/2474], the FNT period 1.6% [46/2891]). A lower incidence of SSIs in patients (odds ratio 0.57, 95% CI 0.36 to 0.88, *P*=0.013), a lower turnover time of the surgical procedure (odds ratio 0.653, 95% CI 0.505 to 0.844, *P*<0.001), and improvement in surgeon satisfaction (odds ratio 1.543, 95% CI 1.039 to 2.292, *P*=0.031), were associated with the FNT period compared with the non-FNT period. However, we did not find significant differences between the FNT period and the non-FNT period in terms of the other indicators.

**Conclusions:**

The presence of an FNT in an OR reduces the incidence of SSIs in surgical patients and the turnover time of surgical procedures and improves surgeon satisfaction. Further implementation of an advanced-practice nurse model with nurse specialists is encouraged.

## Background

Orthopaedic surgery is an important specialty epidemiologically [1], and surgical teams should be capable of ensuring safe surgical procedures, regardless of the setting [2]. The operating room (OR) is a facility equipped for performing surgery. ORs are in high demand; therefore, to maximize OR time, surgical procedures should be organized to provide optimal patient care in a highly efficient manner [3]. OR nurses provide professional nursing in surgery and may play an important role in reducing operation time and improving surgery quality and patient outcomes [3–5]. The work value of nurses is directly reflected in the nursing quality; thus, specific indicators are essential for assessing nursing quality and can provide valuable insights into improving clinical nursing practice [6]. We use the term fixed nurse team (FNT) to represent an OR nurse team in a subdiscipline of surgery that maintains continuity for a long time (at least one year).

OR nurses have reported poor working conditions, including a poor safety climate, teamwork climate, stress level, and perceptions of management [7], which could have an impact on patient outcomes [3, 8–10]. These challenges arise because OR nurses have to contend with multiple demands from patients, family members, surgeons, and other members of interdisciplinary teams [11]. Of the many tasks to complete in a limited period, maintaining effective team communication is important, and researchers have identified standardizing tasks and collecting and using actionable data as cross-cutting themes related to improving team communication [12]. A survey conducted in the Netherlands found that short-term (one-day) fixed teams in bariatric surgery exhibited reduced procedure durations and improved teamwork and safety environment [3]. In addition, as an important component in the development of surgical techniques, specialized skills and training are required for the OR nurse team.

Efficient surgical procedures and safe operations in the OR require high-quality teamwork among professionals [13], and the stability provided by a fixed team may enhance teamwork performance [14]. However, there is a risk of adverse events caused by tension between the conflicting goals of efficiency and safety in the OR [15]. Nevertheless, cooperation and understanding among the surgical team have the potential power to reduce the risks to patient safety [16]. A randomized controlled trial showed that the use of checklists was associated with significant improvements in the management of OR crises [17]. Standardizing tasks, identifying strategies to improve teamwork performance, and ensuring patient safety are important factors for managing the overall nursing quality of the OR team.

There are considerable advantages of fixed teams for surgical procedures. However, several difficulties warrant particular consideration in ORs, and OR nurses may have to perform a large number of surgical tasks in different specializations. Due to increased demand and an insufficient workforce, nurses typically face heavy workloads during their shifts [11]. The deployment of a fixed team should address the concerns identified above by improving efficiency, patient safety and crisis event management [18–20]. However, training and courses on surgical care are not part of the regular curricula at most colleges of nursing [21], and it can be difficult for surgeons to understand the cognitive tasks of an inexperienced nurse during surgery because surgeons and nurses seldom have joint training as a surgical team [13]. Additionally, there is a lack of standardized training, which prevents new nurses from understanding the role of OR nurses during surgical procedures [22, 23]. Moreover, it is a challenge to evaluate the indicators of nursing quality in the OR based on the Centers for Disease Control and Prevention guidelines [24] because of the great heterogeneity among different institutions, which makes it difficult to obtain unanimously agreed upon sensitivity indicators [25]. Currently, there is a serious shortage of OR nurses, and the demand is steadily growing in China [22]. Finally, FNTs are logically feasible and assumed to have advantages in the setting of surgical teams, but only one previous study reported that FNTs did not worsen patient outcomes [3]. Reflective Lifeworld Research recommends the establishment of a fixed team to prevent surgical site infections (SSIs) and ensure patient safety during intraoperative care [5]; additionally, team familiarity has been reported to contribute to reductions in operative time, suggesting potential benefits to maintaining the continuity of team membership over time [26].

The primary aim of this study was to evaluate the effects of FNTs on work efficiency and patient outcomes in orthopaedic surgery ORs.

## Methods

### Study design and participants

A single-arm, nonrandomized trial with a propensity score-matched historically controlled design was conducted from 1 July 2015 to 30 June 2018 to investigate the differences in work efficiency and patient outcomes between an FNT period and non-FNT period in an orthopaedic surgery OR at the Affiliated Hospital of Guangdong Medical University in Zhanjiang China. The hospital has 2223 beds, 43 medical specialties and 4325 employees as well as 21 surgical rooms and 67 OR nurses. General, orthopaedic, gynaecological, otorhinolaryngological, neurosurgical and urological procedures are performed in the OR, and more than 30,000 patients undergo surgical procedures every year.

All patients who underwent orthopaedic surgical procedures and for whom data were available regarding quality nursing indicators during the study period were included, and all nurses who participated in orthopaedic surgical procedures during the two periods were included. The exclusion criteria were documented infection or serious sepsis diagnosed preoperatively in surgical patients, a surgical wound defined as class IV based on the stratified wounds classification system of the National Academy of Sciences [27], and maternity leave or breastfeeding for OR nurses.

### Intervention

The OR nurses worked as administrators (supervisory nursing), professional team leaders, or professional nurses. In the process of management, position responsibilities were formulated to reflect the task value of the nurses in different positions from the point of view of patient-centred care. The FNTs were organized by the professional team leaders, and the team members were professional nurses who worked as a team for at least a one-year period. The task list was distributed to individual team members by the professional team leader each day.

Operative surgical procedures are specialized, precise and complex [28], and it is difficult for OR nurses to fully master the knowledge related to the procedure in a short period. Thus, OR nurses must recognize the complexity of the surgery workflow system and design different work processes to ensure that the FNTs can meet the challenges of a complex adaptive system. We used the pattern of "standardized training", which is hierarchical professional training in different stages. The education secretary of the OR formulated the course content and schedule, and the course content comprised the same topics for different stages based on different mastery level requirements. The training system included 3 years of standardized training and 2 years of OR professional training. The course content included basic system regulations, standardized operating workflows, crisis event management workflows, and specialized surgical procedures, and this content was presented in a dynamic progression for individual nurses in a standardized training period and professional training period. In the FNT period and non-FNT period, the nurses of the standardized training were rotated regularly through different specialized surgeries every six months. After standardized training, in the FNT period, the continuity of the work that the nurses experienced was maintained for at least one year in a given surgical speciality, whereas in the non-FNT period, the nurses were randomly assigned to different surgical specialties according to the OR needs each day.

### Outcome measures

This study used the OR nursing-sensitive quality indicators defined by Li-Hua Huang in China [25], which included 23 indicators with a Kendall W value of 0.147 for importance, 0.051 for the rationality of the calculation formula, and 0.096 for the feasibility of data collection.

The primary outcome of this study was SSIs during the postoperative period in surgical patients [29]. An SSI is defined by the Centers for Disease Control and Prevention of the United States [30] as an infection involving the skin, subcutaneous tissue, deep soft tissues, or any part of the anatomy that was diagnosed as an SSI by a surgeon or attending physician.

The secondary outcome was internal staff job satisfaction (surgeon satisfaction), as surgeon satisfaction correlates with productivity and patient satisfaction [31, 32]. To qualify for inclusion, the surgeon members were required to have performed surgical procedures in the OR. The surgeon survey was administered once by the Quality Assurance Department of the hospital every month and included 10 questions with a maximum score of 100 by no less than ten surgeons. The average of all surgeon scores was used as the monthly satisfaction score, and the informant of the questionnaire was confidential.

According to the study design, the project was implemented from July 2015 to June 2018, including the three steps of the practice process: the baseline survey period (non-FNT period) before implementation (July 2015 to June 2016), the practice change period during the transition period (July 2016 to June 2017), and the post-effect evaluation during the stable period (FNT period) (July 2017 to June 2018). To analyse the effects of the programme on work efficiency and patient outcomes, we compared the measurement indicators between the FNT period and the non-FNT period.

### Data collection

Data were collected from the medical charts database, quality assurance department database, infection control department database, adverse events system database, hospital information system (His) for surgical anaesthesia database, and internal registration database of the OR. The participants whose patients developed SSIs were required to provide the demographic data of the patients, date of operation, and pathogenic microorganisms in both periods.

### Validity and reliability

In this study, we used validation instruments with good reliability to improve the validity and reliability of the data [25]. We had prior experience with nursing management with quality indicators, which were available for the entire data collection period. Quality indicators and procedures were used to maintain uniformity before the study design plan, including surgery-related complications with SSIs, nursing-sensitive quality indicators, and surgeon satisfaction. To eliminate data entry inaccuracies, all the data were inspected carefully before the data were entered, and double entry of data was performed. Data were available from the institutional medical chart database with relevant approval. To control for confounding factors, both the FNT period and the non-FNT period were adjusted for baseline balancing between the two periods by propensity score matching.

### Sample size

In this study, 64 cases (2.6%) of SSIs occurred during the baseline survey period (non-FNT period), and the rate of SSIs was estimated to be reduced by approximately 20% after the stable period with the deployment of FNTs, in which the rate of SSIs was 2.1% per year. Given that this study was an exploratory study based on hypotheses, no attempt was made to estimate the sample size of the study. Instead, all eligible patients in the research unit were enrolled to achieve the maximum statistical power.

### Statistical analyses

Patients in the two periods were classified according to whether they underwent nursing management between the non-FNT period and the FNT period. Categorical variables are presented as numbers and percentages, and continuous variables are summarized as the means (standard deviation [SD]) or medians (interquartile range [IQR]) as appropriate. The primary outcome of the comparative analysis of SSIs in surgical patients and baseline characteristics were compared in surgical patients between the FNT period and the non-FNT period using standardized mean differences (SMDs). Propensity score matching was used to balance the differences in baseline characteristics between patients in the non-FNT period and those in the FNT period. A propensity score, the probability of the FNT period, was estimated using logistic regression based on sex, age, diabetes, hypertension, surgical wound classification, and surgery type [33]. Propensity score matching was implemented using a nearest-neighbour strategy with a minimum calliper of 0.1 [34]. The calliper for the matching was specified in the nearest-neighbour strategy if the unspecified approach did not result in satisfactory balance [35]. To further validate the effects of FNTs, we calculated the odds ratio and 95% confidence interval (CI) by multiple logistic regression analysis, which included the same covariates in patients before and after propensity score matching. As a secondary outcome, in other nursing-sensitive quality indicators, the categorical data were compared between two periods using Fisher’s exact test or the chi-square test as appropriate. The numerical variables were tested for a normal distribution using the Kolmogorov–Smirnov test, and data with *P* values <0.05 were regarded as nonnormally distributed variables. Nonnormally distributed variables were compared using the Mann–Whitney U test, while other variables were compared using t tests between the FNT period and the non-FNT period The tests were 2-sided with a significance level of 0.05. For the odds ratio and 95% CI, the dichotomous logistic regression model was used as appropriate [36]. All statistical analyses were performed using R version 3.6.3 and STATA 15.0.

## Results

A total of 8392 orthopaedic surgeries were performed between 1 July 2015 and 30 June 2018; 439 patients were excluded because of emergency treatment surgery, and 2827 patients were excluded because of changes in the operating surgeon due to the transition period from 1 July 2016 to 30 June 2017. Thus, the sample in our study of the clinical efficacy included 5365 patients: 2474 in the non-FNT period and 2891 in the FNT period. The overall SSI rate was 2.1% (110/5365; the non-FNT period 2.6% [64/2474], the FNT period 1.6% [46/2891]). The baseline characteristics of the surgical patients were comparable between the two periods, and the covariates were well balanced in the propensity score-matched cohort, with all SMDs less than 0.1 (Table 1).

**Table 1:** Baseline characteristics of the surgical patients, according to a nonfixed nurse team and fixed nurse team, before and after propensity score matching

Variable	Unmatched			Matched		
	Nonfixed nurse team (*N*=2474)	Fixed nurse team (*N*=2891)	SMD	Nonfixed nurse team (*N*=2397)	Fixed nurse team (*N*=2397)	SMD
Female, n (%)	1147 (46.4)	1331 (46.0)	0.006	1119 (46.7)	1104 (46.1)	0.013
Age, mean (SD)	56.46 (17.77)	55.82 (18.47)	0.035	56.52 (17.84)	55.80 (17.90)	0.041
Diabetes, n (%)	230 (9.3)	301 (10.4)	0.037	224 (9.3)	196 (8.2)	0.041
Hypertension, n (%)	409 (16.5)	515 (17.8)	0.034	395 (16.5)	383 (16.0)	0.014
Surgical wound classification, n (%) ^a^			0.079			0.018
Class I (clean)	2255 (91.1)	2255 (91.1)		2212 (92.3)	2220 (92.6)	
Class II (clean-contaminated)	101 (4.1)	101 (4.1)		68 (2.8)	69 (2.9)	
Class III (contaminated)	118 (4.8)	127 (4.4)		117 (4.9)	108 (4.5)	
Surgery type, n (%)			0.111			0.017
Bone and Bones	598 (24.2)	747 (25.8)		590 (24.6)	604 (25.2)	
Joints	662 (26.8)	882 (30.5)		661 (27.6)	657 (27.2)	
Spine	909 (36.7)	941 (32.5)		862 (36.0)	865 (36.1)	
Others	305 (12.3)	321 (11.1)		284 (11.8)	276 (11.5)	

*Abbreviation*: *SMD* Standardized mean difference

^a^ The current surgical wound classification system of the National Academy of Sciences, which stratifies wounds into 4 categories (clean, clean-contaminated, contaminated, and dirty), one case of Class IV (dirty) excluded according to exclusion criteria

Table 2 shows that the incidence of SSIs was lower in the FNT period than in the non-FNT period before (odds ratio 0.66, 95% CI 0.44 to 0.99, *P*=0.044) and after propensity score matching (odds ratio 0.57, 95% CI 0.36 to 0.88, *P*=0.013). Regarding the causative pathogens of the SSIs, a total of 29 (26.36%) gram-positive cocci, 63 (57.27%) gram-negative bacilli, and 18 (16.36%) fungi were identified in the two-control period, and we found that the rate of infections with gram-positive cocci was lower in the FNT period than in the non-FNT period before (odds ratio 0.426, 95% CI 0.158 to 1.058, *P*=0.0429) and after propensity score matching (odds ratio 0.332, 95% CI 0.094 to 0.963, *P*=0.025) (Table 3).

**Table 2:** Surgical site infection in the non-fixed nurse team vs. fixed nurse team by multifactor logistic regression analysis

Variable	Unmatched		Matched	
	Odds Ratio (95% CI)	***P*** Value	Odds Ratio (95% CI)	***P*** Value
Fixed nurse team	0.66 (0.44 to 0.99)	0.044	0.57 (0.36 to 0.88)	0.013
Female	0.75 (0.48 to 1.16)	0.211	0.71 (0.43 to 1.14)	0.164
Age	1.00 (0.99 to 1.01)	0.569	1.00 (0.99 to 1.01)	0.760
Diabetes	1.11 (0.57 to 2.03)	0.751	1.07 (0.50 to 2.09)	0.862
Hypertension	0.85 (0.45 to 1.53)	0.613	0.98 (0.49 to 1.82)	0.942
SWC Class I (reference)	1	1	1	1
SWC Class II	5.27 (2.90 to 9.37)	<0.001	5.48 (2.80, 10.42)	<0.001
SWC Class III	7.32 (4.30 to 12.41)	<0.001	7.03 (4.00, 12.39)	<0.001
Bone and Bones (reference)	1	1	1	1
Joints	0.06 (0.01 to 0.20)	<0.001	0.04 (0.00, 0.19)	0.001
Spine	0.23 (0.11 to 0.45)	<0.001	0.24 (0.10, 0.49)	<0.001
Others	1.62 (0.99 to 2.68)	0.056	1.93 (1.12, 3.36)	0.018

*Abbreviation*: *CI* Confidence interval, *SWC* Surgical wound classification system

**Table 3:** Causative pathogens of surgical site infections in orthopaedic surgery between the fixed nurse team period and the non-fixed nurse team period

	Unmatched			Matched		
Causative pathogens	Fixed nurse team period (*N*=2891)	Nonfixed nurse team period (*N*=2474)	*P* Value	Fixed nurse team period (*N*=2397)	Nonfixed nurse team period (*N*=2397)	*P* Value
Gram-positive bacteria	8 (0.28)	16 (0.65)	0.043	5 (0.21)	15 (0.63)	0.025
*Enterococcus gallinarum*	0 (0.00)	3 (0.12)		0 (0.00)	3 (0.13)	
*Enterococcus faecium*	1 (0.03)	2 (0.08)		0 (0.00)	2 (0.08)	
*Staphylococcus aureus*	5 (0.17)	5 (0.20)		4 (0.17)	4 (0.17)	
*Staphylococcus haemolyticus*	1 (0.03)	2 (0.08)		0 (0.00)	2 (0.08)	
*Staphylococcus hominis*	0 (0.00)	1 (0.04)		0 (0.00)	1 (0.04)	
*Streptococcus agalactiae*	1 (0.03)	0 (0.00)		1 (0.04)	0 (0.00)	
*Viridans streptococci*	0 (0.00)	3 (0.12)		0 (0.00)	3 (0.13)	
Gram-negative bacteria	28 (0.97)	35 (1.41)	0.130	21 (0.88)	31 (1.29)	0.163
*Acinetobacter baumannii*	3 (0.10)	7 (0.28)		3 (0.13)	5 (0.21)	
*Enterobacter cloacae*	3 (0.10)	7 (0.28)		2 (0.08)	7 (0.29)	
*Escherichia coli*	4 (0.14)	10 (0.40)		4 (0.17)	8 (0.33)	
*Klebsiella pneumoniae*	8 (0.28)	5 (0.20)		5 (0.21)	5 (0.21)	
*Moraxella catarrhalis*	2 (0.07)	0 (0.00)		1 (0.04)	0 (0.00)	
*Proteus mirabilis*	1 (0.03)	0 (0.00)		1 (0.04)	0 (0.00)	
*Pseudomonas stutzeri*	0 (0.00)	1 (0.04)		0 (0.00)	1 (0.04)	
*Pseudomonas aeruginosa*	5 (0.17)	5 (0.20)		3 (0.13)	5 (0.21)	
*Stenotrophomonas maltophilia*	1 (0.03)	0 (0.00)		1 (0.04)	0 (0.00)	
*Pantoea agglomerans*	1 (0.03)	0 (0.00)		1 (0.04)	0 (0.00)	
Fungi	10 (0.35)	13 (0.53)	0.316	8 (0.33)	13 (0.54)	0.274
*Aspergillus*	0 (0.00)	1 (0.04)		0 (0.00)	1 (0.04)	
*Candida*	5 (0.17)	7 (0.28)		5 (0.21)	7 (0.29)	
*Candida albicans*	1 (0.03)	3 (0.12)		1 (0.04)	3 (0.13)	
*Candida parapsilosis*	1 (0.03)	0 (0.00)		1 (0.04)	0 (0.00)	
*Candida tropicalis*	1 (0.03)	2 (0.08)		1 (0.04)	2 (0.08)	
*Trichosporon*	2 (0.07)	0 (0.00)		2 (0.08)	0 (0.00)	

Table 4 shows that the turnover rate (dichotomous variable: 1=more than 30 minutes; 0=less than 30 minutes) was more efficient in the FNT period than in the non-FNT period (odds ratio 0.653, 95% CI 0.505 to 0.844, *P*<0.001). In addition, the safety climate was determined using the surgeon satisfaction survey designed by the quality assurance department of the hospital. Table 4 shows that surgeon satisfaction was higher in the FNT period than in the non-FNT period (odds ratio 1.543, 95% CI 1.039 to 2.292, *P*=0.031). However, there was no significant difference between the FNT period and the non-FNT period in other nursing qualities, including the rates of checking on the surgical patients, surgery location marks, allergy history, use of antibiotics 60 minutes before incision, expected surgical time, sterilization indicators, surgical equipment and surgical materials availability, surgery name, surgical tools inventory, surgical specimen, postoperative surgical equipment, presence of pressure ulcers during surgery, surgical foreign objects left behind, perioperative drug use, transfusion reaction during the surgical period, unplanned extubation, incidence of needle punctures among medical personnel, incidence of surgical patients falling or falling out of bed, and incidence of electrical burns among surgical patients.

**Table 4:** Secondary outcome of nursing-sensitive quality indicator comparison between the fixed nurse team period and the non-fixed nurse team period

Nursing-sensitive quality indicators	Fixed nurse team period n (%)	Nonfixed nurse team period n (%)	Odds ratio (95% confidence interval)	***χ***^**2**^	***P*** Value
**Patient safety indicators**					
Surgical patient assessment rate, n (%)	2891 (100)	2474 (100)	-	-	-
Surgery location mark assessment rate, n (%)	2891 (100)	2474 (100)	-	-	-
Allergy history assessment rate, n (%)	2891 (100)	2474 (100)	-	-	-
Rate of assessing antibiotics use 60 minutes before incision, n (%)	2891 (100)	2474 (100)	-	-	-
Expected surgical time assessment rate, n (%)	2891 (100)	2474 (100)	-	-	-
Sterilisation indicator results assessment rate, n (%)	2891 (100)	2474 (100)	-	-	-
Surgical equipment and surgical materials availability rate, n (%)	2891 (100)	2474 (100)	-	-	-
Surgery name confirmation rate, n (%)	2891 (100)	2474 (100)	-	-	-
Surgical tools inventory rate, n (%)	2891 (100)	2474 (100)	-	-	-
Surgical specimen checking rate, n (%)	2891 (100)	2474 (100)	-	-	-
Postoperative surgical equipment inspection rate, n (%)	2891 (100)	2474 (100)	-	-	-
Patient acute pressure ulcer rate during surgery, n (%)	5 (0.17)	6 (0.24)	0.715 (0.172 to 2.816)	0.31	0.578
Rate of leaving surgical foreign objects behind, n (%)	0 (0)	0 (0)	-	-	-
Rate of perioperative drug use, n (%)	2891 (100)	2474 (100)	-	-	-
Transfusion reaction rate during the surgical period, n (%)	0 (0)	1 (0.04)	-	1.17	0.280
Unplanned extubation rate, n (%)	2 (0.07)	1 (0.04)	1.712 (0.089 to 101.033)	0.20	0.657
Incidence of needle punctures among medical personnel, n (%)	5 (0.17)	7 (0.28)	0.610 (0.153 to 2.395)	0.72	0.395
Incidence of surgical patients falling or falling out of bed, n (%)	3 (0.10)	2 (0.08)	1.284 (0.147 to 15.382)	0.08	0.784
Incidence of electrical burns among surgical patients, n (%)	1 (0.03)	1 (0.04)	0.856 (0.011 to 67.194)	0.01	0.912
**Efficiency indicator**					
Rate of delaying the operation start time ^a^, n (%)	294 (10.17)	291 (11.76)	0.849 (0.713 to 1.012)	3.48	0.062
Turnover rate of each surgery ^b^, n (%)	123 (9.3)	161 (13.6)	0.653 (0.505 to 0.844)	11.33	<0.001
**Crisis indicators**					
Crisis training completion rate ^c^, n (%)	10 (83.3)	19 (90.5)	0.526 (0.034 to 8.449)	0.37	0.545
**Safety climate**					
Surgeon satisfaction ^d^, median (interquartile range)	81.7 (79.9-86.8)	80 (76.6-80.1)	1.543 (1.039 to 2.292)	4.626	0.031

^a^The denominator is 768 first operations days in each year; ^b^The denominator is 1183 times and 1318 times for the non-fixed nurse team period and the fixed nurse team period, respectively; a positive event is defined as more than 30 minutes for the turnover time between two surgical procedures; ^c^The data are the year-end assessment pass rate in 12 nurses and 21 nurses in the FNT period and the non-FNT period, respectively (≥80, full score 100); ^d^The surgeon satisfaction score was a nonnormally distributed continuous variable, we calculated the odds ratio and 95% confidence interval (CI) by a logistic regression analysis

## Discussion

The results of this study suggest that the nursing management strategy of deploying FNTs in ORs reduced the incidence of SSIs in orthopaedic surgery and the turnover time of surgical procedures and improved surgeon satisfaction with OR nurses. These benefits reduced the apparent incidence of SSIs regardless of the patient's age, sex, diabetes status, hypertension status, wound classification, and surgery type. An evidence-based global perspective reported that the prevention of SSIs is complex and requires the integration of a range of preventive measures before, during, and after surgery [29]; for this purpose, a nursing management strategy involving the use of FNTs better executes the standardized surgical procedures recommended by the WHO as specific to the intraoperative and postoperative periods because every team member knows the sequence of steps during surgery and is better able to think ahead [3]. An observational study reported that a single surgical team seems to provide protection against postoperative SSIs [9]. The advantage of working in a team with long-term stability includes improved confidence of the surgeon, which results in better performance; surgical teams should engage all members of the surgical ecosystem [10], and the safety environment is better in the context of good teamwork.

This study also found that the turnover time of the surgical procedure was shorter in the FNT period than in the non-FNT period. The turnover time between one patient leaving the OR and the next patient arriving (the start of the incision of the next surgery) can be directly observed in the nursing records of medical charts, and this approach to data collection can reduce the occurrence of hidden problems [25]. Reducing the turnover time of the surgical procedure is an important aspect of a multifaceted solution to increasing efficiency. An observational study reported that orthopaedic-specific staff can reduce turnover time [37]. An accountable nurse team demonstrated a reduction in surgical turnover time [38]. Therefore, a higher efficiency in the nurse team and a good safety climate in the surgical team lead to better outcomes for patients.

Our data show no significant difference between the FNT period and the non-FNT period in other nursing-sensitive quality indicators. For example, crisis training is required to be 100 percent completed by every nurse who works in the OR, and we used the pass rate in the annual assessment to evaluate crisis event management ability; thus, the crisis training completion rate may not truly reflect changes in nursing quality [25]. Similarly, other unmeasured confounding factors may have been nursing-sensitive quality indicators.

### Study implications

More explicit OR nursing management and more direct outcomes were observed, which is a strength of the study. However, this study also has several limitations. First, this study was not a randomized trial; thus, the study results may need further verification by a high-quality randomized trial [39]. Second, we could not evaluate the procedure durations of each surgery using reliable measures. We believe that single-item measures of surgical efficiency have validity to some extent. Although we did match the type of surgery at baseline, we acknowledge that the reliability of the measures should be interpreted cautiously due to the possible heterogeneity of the surgeons in this study [40]. Third, this was a single centre study. Finally, we did not find a significant difference in many indicators between the FNT period and the non-FNT period. We believe that the measures of surgical efficiency examined in this study are valid to some extent. However, there was no significant difference in these indicators, which emphasizes the necessity of quality indicators of patient safety in all periods and that these indicators, such as the rate with which surgical patients are checked, must be met 100% of the time. Finally, some issues emerged after the implementation of FNT; notably, the fixed team of nurses was not completely separated from the non-FNT nurses due to variations in the number of surgeries performed in each specialty each day, and some nurses may not be well adapted to the continuous development of cross-specialty surgical procedures. Thus, a randomized controlled trial is needed to verify the causal relationship identified in the current study.

## Conclusions

The nursing management strategy involving the deployment of FNTs in the OR reduces turnover time and the incidence of SSIs in orthopaedic surgery and improves surgeon satisfaction. Whether the use of an FNT is effective in other surgical settings requires further research. The findings of this study contribute to leadership development for OR nurses and leaders and improve patient outcomes and can improve surgery service delivery and guide hospital policies and reforms. OR nurses need more specialized training and practice to adapt to developing surgical techniques, and further deployment of nurse specialists with advanced practice nurse models is encouraged.

## Data Availability

Data are available from the institutional medical chart database with relevant approval. The datasets used and/or analysed during the current study are available from the corresponding author on reasonable request.

## Abbreviations

OR: Operating room
FNT: Fixed nurse team
SSIs: Surgical site infections
SD: Standard deviation
IQR: Interquartile range
SMDs: Standardized mean differences
CI: Confidence interval
